# Dr. Frederick H. James

**Published:** 1897-08

**Authors:** 


Dr. Frederick H. James, of Lancaster, died at his home in that village, June 28, 1897, aged 72 years. He retired from professional work some years ago, but retained the presidency of the glass works at Lancaster and was a trustee of the Erie County Savings Bank until his death.

Dr. James's gift to the Buffalo Fine Arts Academy of a rich collection of line engravings and etchings is reckoned, for its general value, as one of the best in the country ; and in its representation of the work of Seymour Haden it is unsurpassed anywhere. It is stated that the collection includes an example—often in choice proof state—of every known work of this great master of etching.

His gift of rare coins to the Buffalo Historical Society was equally noteworthy. There are over 13,000 pieces, representing probably more than that number of dollars, and including many coins, tokens, medals and the like of great rarity and historic interest. He was one of the best connoisseurs on the subject in this country and used to declare that there were many pieces in his collection which, if lost or destroyed, could never be replaced.

Dr. James had been a corresponding member of the Historical Society since 1869, a life member since 1890 and a member of the board of managers since 1894. At a meeting of the board recently a suitable memorial was adopted and the board voted to attend the funeral in a body.

